# The NS1 Glycoprotein Can Generate Dramatic Antibody-Enhanced Dengue Viral Replication in Normal Out-Bred Mice Resulting in Lethal Multi-Organ Disease

**DOI:** 10.1371/journal.pone.0021024

**Published:** 2011-06-22

**Authors:** Andrew K. I. Falconar, Fernando Martinez

**Affiliations:** 1 Grupo de Investigaciones en Enfermedades Tropicales, Departmento de Ciéncias Básicas Médicas, Universidad del Norte, Barranquilla, Colombia; 2 Department of Pathogen Molecular Biology, London School of Hygiene and Tropical Medicine, London, United Kingdom; 3 The University of Texas M.D. Anderson Cancer Center, Houston, Texas, United States of America; Global Viral Forecasting Initiative, United States of America

## Abstract

Antibody-enhanced replication (AER) of dengue type-2 virus (DENV-2) strains and production of antibody-enhanced disease (AED) was tested in out-bred mice. Polyclonal antibodies (PAbs) generated against the nonstructural-1 (NS1) glycoprotein candidate vaccine of the New Guinea-C (NG-C) or NSx strains reacted strongly and weakly with these antigens, respectively. These PAbs contained the IgG2a subclass, which cross-reacted with the virion-associated envelope (E) glycoprotein of the DENV-2 NSx strain, suggesting that they could generate its AER via all mouse Fcγ-receptor classes. Indeed, when these mice were challenged with a low dose (<0.5 LD_50_) of the DENV-2 NSx strain, but not the NG-C strain, they all generated dramatic and lethal DENV-2 AER/AED. These AER/AED mice developed life-threatening acute respiratory distress syndrome (ARDS), displayed by diffuse alveolar damage (DAD) resulting from i) dramatic interstitial alveolar septa-thickening with mononuclear cells, ii) some hyperplasia of alveolar type-II pneumocytes, iii) copious intra-alveolar protein secretion, iv) some hyaline membrane-covered alveolar walls, and v) DENV-2 antigen-positive alveolar macrophages. These mice also developed meningo-encephalitis, with greater than 90,000-fold DENV-2 AER titers in microglial cells located throughout their brain parenchyma, some of which formed nodules around dead neurons. Their spleens contained infiltrated megakaryocytes with DENV-2 antigen-positive red-pulp macrophages, while their livers displayed extensive necrosis, apoptosis and macro- and micro-steatosis, with DENV-2 antigen-positive Kuppfer cells and hepatocytes. Their infections were confirmed by DENV-2 isolations from their lungs, spleens and livers. These findings accord with those reported in fatal human “severe dengue” cases. This DENV-2 AER/AED was blocked by high concentrations of only the NG-C NS1 glycoprotein. These results imply a potential hazard of DENV NS1 glycoprotein-based vaccines, particularly against DENV strains that contain multiple mutations or genetic recombination within or between their DENV E and NS1 glycoprotein-encoding genes. The model provides potential for assessing DENV strain pathogenicity and anti-DENV therapies in normal mice.

## Introduction

Dengue viruses (DENVs), which occur as four discrete serotypes, are the most important vector-borne human viruses [1]. Dengue hemorrhagic fever and dengue shock syndrome (DHF/DSS), which are the most severe forms of disease were previous classified into four grades (DHF I to IV) [2], but have now been re-classified through a TDR/WHO program [3], in which human ‘severe dengue’ cases that require urgent emergency treatment have been characterized by: i) severe plasma leakage leading to dengue shock and/or fluid accumulation with respiratory distress, ii) severe hemorrhages, or iii) severe organ impairment (hepatic damage, renal impairment, cardiomyopathy, encephalopathy or encephalitis) [3]. DHF/DSS cases result from the over-activation of patients' immune responses, usually during secondary DENV infections with virulent heterologous DENV serotypes [4]. The severity of clinically-graded DHF/DSS directly correlated with the plasma levels of the complement anaphylotoxins (C3a and C5a), histamine, particular cytokines (e.g. IFN-γ, TNF-α, IL-1, IL-6 and IL-10), and chemokines (e.g. IL-8 and MIP-1), with increased clearance of the C1q (complement) glycoprotein [5], [6]. Numerous studies have shown that IgG antibodies generated against the DENV virion-associated envelope (E) and pre-membrane (prM) glycoproteins can increase DENV replication in Fcγ receptor (FcγR)-bearing cells *in vitro* when they are diluted beyond their effective neutralizing titers [4]. Some monoclonal antibodies (MAbs), however, generated enhanced disease in mice when they were administered before challenge with other flaviviruses, but without increased viral replication [7], [8]. The terms antibody-enhanced replication (AER) and antibody-enhanced disease (AED) were, therefore, proposed to clarify these different *in vivo* findings [9], both of which were previously described as antibody-dependent enhancement (ADE) [7], [8]. The greatest DENV AER was, however, obtained *in vitro* using undiluted polyclonal antibodies (PAbs) obtained from children during the acute-phase of DENV infections that subsequently developed DHF/DSS [10], or at the age when most DHF/DSS cases occurred [11]. Despite these findings and their importance for understanding of DENV pathogenesis, the ability of undiluted PAbs raised against DENV to subsequently generate AER of a heterologous DENV serotype *in vivo* was assessed in only one study [12]. In this study, approximately 50-fold increased DENV-2 titers, and longer durations of viremia were observed in monkeys, but they did not develop disease symptoms [12]. DHF/DSS patients generated much higher titers of DENV-specific antibodies of the IgG1, than IgG2, subclasses during the acute-phase of disease compared to those from DF patients [13], and which could generate DENV AER in both FcγRI- and FcγRII-bearing cells [14], [15]. Antibodies of the human IgG1 and mouse IgG2a subclasses are similar since they are stimulated by IFN-γ, fix complement and recruit ADCCs, and are uniquely bound by all four FcR types, while those of the human IgG2 and mouse IgG1 subclasses are not stimulated by IFN-γ, do not fix complement or recruit antibody-dependent cytotoxic cells (ADCCs), and are only bound by low affinity FcRs [16]–[18]. As such, the ability of DENV E glycoprotein-specific antibodies to either neutralize or generate AER was dependent on their ability to fix complement and, therefore, their IgG subclasses [19].

The dengue virus nonstructural-1 (NS1) glycoprotein, which provided Fc-dependent non-neutralizing antibody-mediated protection in animals [20], [21], is a candidate vaccine proposed to avoid the risk of DENV AER posed by generating unsustainable neutralizing IgG titers against the virion-associated E/prM glycoproteins [22]. We however previously showed that some purified MAbs (e.g. MAb 1G5.3) generated against the DENV-2 NS1 glycoprotein, also cross-reacted with common epitopes on the DENV envelope (E) glycoproteins, weakly neutralised them [23], and also generated a dramatic DENV-2 antibody-enhanced replication (AER) resulting in lethal antibody-enhanced disease (AED) in mice [9]. In addition, MAb, 1G5.4-A1-C3 and mouse PAbs generated against the DENV-2 NS1 glycoprotein in out-bred (TO strain) or congeneic (H2 class II: B10 strain) mice, all cross-reacted with human fibrinogen, platelets and endothelial cells [24], [25]. These PAbs generated by the low-responding (H2^d^: B10.D_2_N and BALB/c strains) mouse haplotype or the low-avidity MAb 1G5.4-A1-H6 subclone, and the high-responding (H2^s^: B10.S strain) mouse haplotype or the high avidity MAb 1G5.4-A1-C3 subclone, showed similar reaction patterns against the immuno-dominant ELK/KLE-type epitopes as those generated by DF and DSS patients, respectively [24], [25]. In addition, MAb 1G5.4-A1-C3 more strongly reacted with the ELK/KLE-type epitopes on the E glycoproteins of virulent (DHF/DSS-associated) DENV-2 and DENV-3 strains [23]. Such PAbs were, therefore, thought to play important roles in DENV AED, but which would be dependent upon: a) the concentrations of their IgG subclasses which could fix complement and recruit ADCCs, b) their relative avidities for these epitopes on their DENV NS1 and E glycoproteins, c) the relative concentrations of their DENV NS1 and E glycoproteins, d) the relative concentrations of fibrinogen, platelets, endothelial cells or other auto-antigens against which they cross-react, and d) the presence of complement, IFN-γ and ADCCs. Thus, we consider it to be absolutely essential to perform these studies *in vivo* using undiluted PAbs.

The DENV titers present in different organs from DHF/DSS patients have not yet been determined. Comparative studies have, however, been performed on different organs from fatal DSS cases using histo-pathology [5], [26], DENV-isolation efficiencies [5], [26], *in situ* hybridization [27], and DENV-specific MAbs [27]–[30]. In these studies, macrophages in their spleens, lungs and livers contained DENVs or their antigens [5], [24]–[28], as well as their brain phagocytic microglia and astrocytes [28], [29], [31]. The spleen and liver were major sites of DENV replication [27], and dramatic DENV AER was generated in primary splenic macrophages (red pulp), but not T or B cells (white pulp), using DHF/DSS patients' PAbs *in vitro* [32]. DENV has frequently been isolated from DHF/DSS patients' livers [5], [26], and histological analyses demonstrated severe liver damage characterized by steatosis, necrosis and apoptosis (pyknosis), with DENV antigen-positive Kuppfer cells and hepatocytes [33], [34], similar to, but less severe than, that caused by yellow fever virus [35].

The great majority of these fatal DHF/DSS cases demonstrated sufficiently severe lung histo-pathology to hinder gaseous exchange which, therefore, contributed to hypoxia and metabolic acidosis [26]. In a very large study of DHF/DSS autopsies, 85/100 (85%) of them displayed dramatically increased infiltrations of mononuclear cells and megakaryocytes, with edematous septa containing eosinophic precipitates [26]. These results, therefore, demonstrated that these lung pathologies observed in the majority (85%) of these patients [26] were not due to therapeutic fluid overload, which may also lead to respiratory distress [3]. Acute respiratory failure was also reported to be the leading cause of death in DSS patients who succumbed after plasma leakage was resolved [26]. Importantly, acute respiratory distress syndrome (ARDS) has subsequently been reported to occur in patients with the most severe DENV disease grades (DSS: DHF grades III and IV), was a major cause child and adult deaths in some studies, and occurred even when appropriate early hospital-based supportive therapy was provided [36]–[39]. DENVs have increasingly been implicated in causing ARDS [37], DSS was identified as the third most common cause of ARDS in one study [36], and symptoms of ‘fluid accumulation with respiratory distress’, have now been added as symptoms of ‘severe dengue’ by the TDR/WHO steering committee [3]. DSS-associated ARDS, as with other acute viral diseases, occurs through the dramatic and diffuse alveolar damage (DAD) due to edema and the infiltration of mononuclear cells, which result from the excessive release of IFN-γ and other inflammatory mediators [26]. DSS-associated ARDS may also cause multi-organ dysfunction syndrome (MODS) and disseminated intravascular coagulation (DIC), due to resultant metabolic acidosis, which were also causes of DSS-associated mortalities [36]–[39], as well as neurological disease [38]. Although rarer, DHF/DSS-associated encephalopathy, myelitis, meningitis and encephalitis, have been increasingly reported throughout the world, and in some reports encephalopathy [40], myelitis or encephalitis [41] were associated with poor prognoses. DENVs were the leading cause of encephalitis (47%) in one DENV-endemic country (Brazil) [42], caused 7% (28/401) of the encephalitis cases amongst those with suspected viral CNS infections in a study conducted in Jamaica [43], 7% in Indonesia [44], and the third highest cause of viral encephalitis (4.6%) in a study conducted in Viet Nam, where Japanese encephalitis virus was prevalent [45]. As a result, both DENV encephalopathy and encephalitis are now classified as criteria of ‘severe dengue’ in humans by the TDR/WHO steering committee [3].

Traditionally, the normal route of assessing both active and passive protection against DENV infections in animals has been by the intra-cerebral challenge of mice [46], but this challenge route is still extensively used for these purposes, and also to confirm the attenuation of candidate DENV vaccines. This model has, therefore, also been used to test the protective roles of non-neutralizing antibodies generated against DENV NS1 glycoproteins [47]–[50], and capsid (C) proteins [51], [52]. Despite using this challenge route, the type and quantity of DENV antigens subsequently observed in the brains and livers of these mice were similar [53], with both DENV structural (C and E) and non-structural (NS1) proteins detected in them [54]. These results, therefore, indicated that the DENV spread to their peripheral organs when their blood-brain barrier was breached.

In this study, we tested whether PAbs raised against the DENV-2 NS1 glycoprotein could generate AER of two DENV-2 strains, one of which possessed a less antigenic NS1 glycoprotein, and resulted in AED in out-bred mice under normal physiological conditions. The virological findings were then supported by comparative histo-pathological and immuno-histological studies on their lungs, brains, spleens and livers, and also with those reported in DSS patients. In addition we: 1) assessed whether detectable IgG2a antibodies were generated in these mice, 2) attempted to block the AED with high concentrations of the NS1 glycoprotein, and 3) attempted to isolate the DENV-2 from samples of lung, spleen and liver of these mice.

## Materials and Methods

### Ethics statement

All animal experiments adhered to UK Home Office regulations, in accordance with the UK Animals (Scientific Procedures) Act 1986, were performed in approved animal facilities under relevant project and personal animal procedure licenses (PIL 70/6903), and were approved by the London School of Hygiene and Tropical Medicine (LSHTM) ethics committee. These animal experiments also conformed to European guidelines (European Convention for the Protection of Vertebrate Animals used for Experimental and other Scientific Purposes: Council Directive 86/609/EEC).

### Dengue virus growth *in vitro*

Low passage DENV-2 of the New Guinea-C (NG-C) prototype strain and the NSx (NSx) strain, which was a putative American/Asian genotype strain that possessed a less antigenic NS1 glycoprotein due to either amino acid substitutions or genetic recombination, were obtained from John Aaskov (Queensland Institute of Technology, Brisbane, Australia) and Colin Leake (LSHTM, London, UK), respectively. Both of these DENV-2 strains were isolated from DF patients, they were specifically identified by the DENV-2- E glycoprotein-specific MAb 3H5, and limited cDNA sequence determination confirmed that they both encoded the non-American DENV-2 genotype 390-asparagine (N) residue in their E glycoproteins [55]. The growth of dengue viruses in mammalian (Vero) cells, insect (C6/36) cells and in suckling mouse brains were performed as described previously [23], [25], [56]. Viruses were cultured in Vero cells maintained in complete medium 199 (MCGM: mammalian cell growth medium) and C6/36 cells maintained in complete Leibovitz L-15 medium (ICGM: insect cell growth medium). Cell-culture supernatants were collected on day 4 and 8 after infection.

### Purification of the DENV-2 virions and NS1 glycoproteins

The purification of DENV-2 virions from infected mammalian (Vero) and insect (C6/36) cell-culture supernatants and the immuno-affinity purification of the native multimeric form of the DENV-2 extracellular/secreted (e/sNS1) glycoprotein from the supernatants of infected mammalian cells were performed as described previously [23]–[25]. For this study, polyethylene glycol (PEG) precipitated DENV-2 virions from infected insect cell-culture supernatants were purified by ultra-centrifugation on sequential 20/50% (wt/wt) discontinuous and 20–50% (wt/wt) continuous sucrose gradients. Fractions containing the purified DENV-2 virions were then diluted and the DENV-2 virions re-precipitated using PEG and, after centrifugation, they were re-suspended in the minimal volume of buffer and stored at −80°C.

The native extracellular/secreted forms of DENV-2 NS1 (e/sNS1) glycoproteins were obtained from mammalian (Vero) cell cultures infected with the DENV-2 (NG-C and NSx strains) and maintained in medium 199 containing 3.5% foetal bovine serum and antibiotics as described previously [24], [25]. The supernatants were then collected 4 and 8 days after infection, a cocktail of protease inhibitors was added, they were made to 30 mM Tris/HCl (pH 7.4), 0.02% (wt/vol) NaN_3_ containing 7% (wt/vol) PEG 8,000 (P2139, Sigma) with 0.4 M NaCl, and the DENV was allowed to aggregate overnight at 4°C. Mammalian (BHK) cells, which stably expressed the DENV-2 (16681 strain) NS1 glycoprotein [57], was kindly provided by Michael Diamond (Washington University School of Medicine, St. Louis, USA), and maintained in RPMI medium containing 10% FBS and 3 µg/ml puromycin (P8833, Sigma), and the supernatants were harvested when the cells reached 90% confluent. Protease inhibitors were then added, and they were made to 30 mM Tris/HCl (pH 7.4) with 0.02% (wt/vol) NaN_3_. These supernatants were then clarified by centrifugation at 8,000 xg, and slowly (1 ml/min) passed through an immuno-affinity column containing 12 mg of MAb 2A5.1. After washing, the bound DENV-2 NS1 glycoproteins were eluted in their native hexameric forms using 20 mM diethylamine/PPB (pH 11.2), and fractions were immediately neutralized as described previously [57], [58]. Protein concentrations were determined against standard concentrations of bovine serum albumin (BSA) by the microtiter plate-adapted bicinchoninic acid (BCA) protein assay (Pierce, USA).

### Dengue virus growth in mice

Pathogen-free out-bred Tyler's original (TO) mice, which were previously used for active and passive protection experiments against DENVs [9], [56], were employed to test whether polyclonal antibodies (PAbs), generated against immuno-dominant epitopes on the DENV-2 NS1 glycoprotein [23]–[25] which cross-reacted with these determinants on the DENV E glycoproteins [23], could all generate DENV-2 AER/AED *in vivo*, as was initially reported [9]. For this study, the DENV-2 strains were grown once in 1–2 day old pathogen-free out-bred suckling TO mice (Harlan-OLAC, UK) as described [56] by injecting 10 µl of DENV-2-infected supernatant by the intra-cerebral (i–c.) route under anaesthesia using 3% (vol/vol) halothane (Rhone Merieux, Ireland) in oxygen at 1 dm^3^/min. When mice showed severe neuro-pathological symptoms, they were killed by CO_2_ asphyxiation, frozen to –80°C, thawed, and their brains were aseptically harvested. Foetal bovine serum (50% vol/vol) in MCGM was added and brain homogenates were prepared, clarified by centrifugation, the clarified 10% (wt/vol) DENV-2-infected mouse brain extracts were collected, and aliquots were stored at –80°C.

To determine the DENV-2 NG-C and NSx strain challenge doses used in the subsequent DENV-2 AER/AED experiments, four groups of 10 six week-old pathogen-free out-bred TO mice (Harlan-OLAC, UK) were anaesthetised and challenged by the intra-cerebral (i–c.) route with 40 µl of serial 10-fold dilutions of the 10% (wt/vol) DENV-2-infected mouse brain homogenates prepared in sterile RPMI-1640 medium. Severe morbidity in which the mice showed severe respiratory distress and hind-leg paralysis, which were classified as mortalities, were recorded daily and when these mice were humanely killed. The dilution of DENV-2 NG-C or NSx strains that caused 25% mortality (0.5 LD_50_) was then determined.

For the DENV-2 AER/AED experiments, groups of 14-16 three-week old pathogen-free out-bred TO mice (Harlan-OLAC, UK) were immunized with 10 µg of immuno-affinity purified multimeric e/sNS1 glycoproteins of the DENV-2 NSx (group A), NG-C (groups B and C), or ovalbumin (control protein) (group D) emulsified in Freund's complete adjuvant (FCA) by combined intra-peritoneal (i-p.)/sub-cutaneous (s-c.) routes. Two weeks later, each mouse was boosted with the same antigen dose contained in PBS by the i-p. route. One week later (i.e. at six-weeks old), each mouse was anaesthetised, a blood sample was obtained from their retro-orbital sinus for the ELISA and PRNT assays, and they were challenged by the i-c. route with 40 µl containing less than 0.5 LD_50_ (approximately 0.5 to 1.0×10^3^ pfu) of either the DENV-2 NSx (groups A, B and D) or NGC (group C) strains. Severe morbidity, displayed as severe respiratory distress and hind-leg paralysis, was classified as mortality, and was recorded over a 14 day period after which time no further ‘mortalities’ occurred; when observed such mice were humanely killed using CO_2_. For the DENV-2 AER/AED blocking experiments, three groups (A, B and C) of 14–16 three-week old pathogen-free out-bred TO mice (Harlan-OLAC, UK) were immunized with 10 µg of immuno-affinity purified multi-meric e/sNS1 glycoprotein of the DENV-2 NG-C strain emulsified in FCA by combined i-p./s-c. routes. Two weeks later, each mouse was boosted with the same antigen dose contained in PBS by the i-p. route. One week later (i.e. at six-weeks old), 250 µl of sterile protein-free RPMI medium (negative control) was administered intra-cerebrally to each mouse in group A immediately prior to, and at the same site as, the challenge with 40 µl containing <0.5 LD_50_ (approximately 0.5 to 1.0×10^3^ pfu) of the DENV-2 NSx strain. At the same time, each mouse in the other groups received 500 µg of the purified native multi-meric forms of the DENV-2 e/sNS1 glycoprotein of either the NSx (group B) or NGC (group C) strains contained in 250 µl of protein-free RPMI-1640 0.2 µm filter-sterilized medium, by the i-c. route immediately prior to, and at the same site as, the challenge with 40 µl containing <0.5 LD_50_ of the DENV-2 NSx strain. Severe morbidity, displayed as severe respiratory distress and hind-leg paralysis, was classified as mortality, and was recorded over a 14 day period after which time no further ‘mortalities’ occurred; when observed such mice were humanely killed using CO_2_. Kaplan-Meier survival curves were used for statistical comparisons between these different mouse groups using MedCalc statistical software version 11.3 ( http://www.medcalc.be/ ).

A group of 5 three-week old out-bred TO mice were also immunized using the same dose, adjuvant and routes with the immuno-affinity purified DENV-2 (16681 strain) NS1 glycoprotein from the BHK replicon.

Positive control mouse PAbs for the immunoblot assays were prepared in a group of three-week old out-bred TO mice immunized every three weeks with approximately 2×10^5^ pfu of live DENV-2 (NG-C strain) by the i-p. route before blood was collected and the sera stored at −80°C.

### Mouse tissue samples

Brains were aseptically collected from some of the mice 14 days after challenge with the DENV-2 NSx strain, weighed and 10% (wt/vol) DENV-2-infected mouse brain homogenates were prepared as described above. The DENV-2 titres were determined by plaque assays in 48-well cell-culture plates (Costar, USA). For these assays, serial 10-fold dilutions from 1/10 were prepared in 250 µl of MCGM and 250 µl of MCGM containing 2×10^5^ Vero cells/well were added. Each well was subsequently overlaid with 500 µl of 1.5% (wt/vol) carboxy-methylcellulose (C5678, Sigma) prepared in MCGM. The wells were incubated for 7 days, fixed with 8% (wt/vol) formaldehyde, washed, stained with 0.01% (wt/vol) crystal violet in PBS, washed again with H_2_O, and air-dried. The average DENV-2 titers (plaque forming units/gram of mouse brain material) were determined by multiplying the average numbers of plaques/well x 4 ( =  plaques/ml) x the log_10_ dilution x 10. In addition, lungs, spleens and livers from the DENV-2 AER/AED mouse group (n = 4) which died 8–9 days after DENV-2 NSx strain challenge were collected after storage of these mice at −80°C. These tissues were aseptically teased and homogenised in ICGM using 3 ml mini-glass tissue homogenizers (GP/20402, Camlab, UK), clarified by centrifugation and the supernatants were used to infect 25 cm^2^ C6/36 insect cell monolayers. After incubation at 28°C for 7 days, the supernatants were collected and clarified by centrifugation. After discarding the supernatants, the cells were re-suspended in a minimum volume of PBS added to 12-well polytetrafluoroethylene- (PTFE-) coated immuno-fluorescent slides (Hendley, UK), air-dried, fixed with cold (-20°C) acetone, again air-dried and stored at −20°C (see immunoassays).

### Histological studies

Mouse brains, lungs, spleens and livers were aseptically collected on day 14 after virus challenge, placed on 2 cm diameter cork discs and covered with Tissue Tek OCT compound (PELCO International, USA), slowly frozen and stored at −80°C. Six µm tissue sections were cut using a cryotome and placed on slides which were fixed with cold (−20°C) acetone, ethanol and again with acetone, air-dried and stored at –80°C. Some of these slides were stained using standard iron hematoxylin and eosin (H&E). Alternatively, the sections were wetted with PBS, before a 1/1000 diluted pool of DF patients sera that had a high ELISA titer (mean ELISA reciprocal log_10_t_50_ 5.83) against the E glycoproteins on purified DENV-2 (NSx strain) virions, but reacted very weakly with its NS1 glycoprotein (mean ELISA reciprocal log_10_t_50_ 2.25), were reacted with the sections for 1 hr at 25°C. After washing with PBS, A FITC-labelled goat anti-human IgG (H and L) (109-095-088: Jackson ImmunoResearch, USA), diluted at 1/1000 containing 0.03% (wt/vol) Evan's blue, was then reacted with the sections for 1 hr at 25°C. After washing with PBS, and briefly (3 secs) in H_2_O, they were mounted in 90% glycerol/PBS pH 8.0. Photomicrographs were taken for 4–6 mins using the appropriate excitation and barrier filters on Fujichrome Sensia 400 film (Fuji Inc. Japan) and subsequently converted to digital format. For these studies, megakaryocytes and the DENV-2 target tissue macrophages in the liver (Kuppfer cells), lungs (alveolar macrophages), spleen (red-pulp macrophages) and brain (microglia cells) were identified by their characteristic morphologies according to i) mouse histological atlases [ http://ctrgenpath.net/static/atlas/mousehistology/Windows/introduction.html , http://www.deltagen.com/target/histologyatlas/HistologyAtlas.html , http://tvmouse.compmed.ucdavis.edu , http://www.mbl.org/atlas170/atlas170_frame.html ], ii) histo-pathological descriptions and photo-micrographs from patients with DENV acute respiratory distress syndrome (ARDS) [5], [26], [59], iii) megakaryocyte infiltration of patients' organs during DENV infections [5], [26] or in the spleens of genetically modified (knockout) mice [60], and, iv) DHF/DSS patients' liver samples [5], [26], [33], [34] that showed DENV- infected Kuppfer cells [61]. The histological findings in the brains of the DENV AER/AED mice were also compared with encephalitis in humans or mice caused by West Nile virus [62], Saint Louis encephalitis virus (slide 109) [ http://www.urmc.rochester.edu/neuroslides ] and Japanese encephalitis virus [63].

### Immunoassays

The indirect ELISAs and immunoblot assays, using the immuno-affinity purified native hexameric DENV-2 e/sNS1 glycoproteins and purified DENV-2 virions, were performed as described previously [23]. After loading the ELISA plates at either 0.6 µg/ml (purified DENV-2 virions) or 1.5 µg/ml (purified DENV-2 NS1 glycoproteins), they were blocked using 1% (wt/vol) gelatin in PBS. After PBS washing, serial PAb or MAb dilutions were reacted. After washing, the bound PAbs were detected by sequential reaction steps using a peroxidase-labelled goat anti-mouse IgG (H & L) (115-035-062, Jackson ImmunoResearch diluted to 1/2000, washing, and addition of standard *o*-phenylenediamine dihydrochloride substrate solution containing H_2_O_2_. After stopping the reaction with 0.2M H_2_SO_4_, the absorbance values were measured at a dual wavelength of 490 and 620 nm (MRX, Dynex) and the average reciprocal log_10_ 50% end-point ELISA titers (1/log_10_t_50_) were determined.

The plaque-reduction neutralisation tests (PRNTs) were performed in 48-well plates using serial dilutions of both the DENV-2 NG-C and NSx strains from infected C6/36 supernatants subsequently diluted in MCGM against serial pre-challenge sera from mice and 2×10^5^ Vero cells/well were performed as described previously [23], [56]. These wells were then overlaid with 1.5% (wt/vol) carboxy-methylcellulose/MCGM and after incubation at 37°C for 7 days the cell monolayers were fixed with 8% formaldehyde, washed and stained with 1% (wt/vol) crystal violet/PBS before further being washed and dried (see above). The PAb dilutions which reduced the numbers of DENV-2 plaques by 50% were then calculated.

To investigate the possible contamination of the immuno-affinity purified DENV-2 e/sNS1 glycoprotein samples with the DENV E and prM glycoproteins, high (960 ng) concentrations of the purified DENV-2 (NSx strain) virions also obtained from DENV-infected mammalian cells and 200 ng concentrations of the purified e/sNS1 glycoproteins of the DENV-2 16681, NG-C and NSx strains were heated and subjected to 8% (wt/vol) non-reducing SDS-PAGE. To identify the cross-reaction of PAbs generated against the purified DENV-2 e/sNS1 glycoproteins of the 16681, NG-C and NSx strains two (approximately 1000 ng and 250 ng) concentrations of DENV-2 virions purified from DENV-2 (NSx strain) infected C6/36 cell-culture supernatants (see above) were heated at 100°C for 3 min before subjection to 9% (wt/vol) non-reducing SDS-PAGE. These gels were then subjected to semi-dry electro-blotting onto 0.2 µm pore-sized nitrocellulose membranes and air drying. After blocking with PBS/M (see immuno-fluorescent assays), a 1/200 dilution of the mouse or human PAbs, or 1 µg/ml of the IgG2a subclass MAb, specific for the DENV-2 NS1 (MAb 2A5.1), E (MAb 2C5.1) or prM (MAb 2A4.1) glycoproteins [23], were reacted with these membranes. After washing, the bound PAbs were detected by sequential reaction steps using a 1/2000 dilution of the peroxidase-labelled anti-mouse IgG2a-specific second PAbs (115-035-206, Jackson ImmunoResearch, USA), washing and standard 3,3′ diaminobenzidine tetrahydrochloride/4-chloro-1-naphthol (CND) substrate mixture containing H_2_O_2_.

Immuno-fluorescent antibody (IFA) assays to detect DENV-2 infected C6/36 cells were performed as described previously [64]. For these assays, MAb 3H5 specific for the envelope (E) glycoprotein DENV-2 was diluted to 1/100 in PBS containing 2% milk powder (Marvel, Cadbury's, UK) and reacted with the fixed C6/36 cells on the IFA slides for 2 hr at 28°C. These slides were then washed three times with PBS and gently blotted before adding 10 µl of a 1/500 dilution of FITC-labeled goat anti-mouse IgG (H&L) (115-095-062, Jackson ImmunoResearch, USA) and incubated at 28°C for 1 hour. After washing again three times with PBS, the slides were briefly dipped in distilled water, gently blotted and mounted with 90% glycerol/PBS pH 8.3 and viewed under immuno-fluorescent microscopy using the appropriate FITC excitation and barrier filters.

## Results

### Affinity purified DENV NS1 glycoproteins did not contain other DENV proteins

Since the NS1 glycoproteins were immuno-affinity purified from DENV-2 infected mammalian cells, we initially tested whether these preparations contained any contaminating DENV-2 E or prM glycoproteins, which could affect the results. We also used a control immuno-affinity purified NS1 glycoprotein recombinant expression construct which expressed the NS1 glycoprotein of DENV-2 (16681 strain) in mammalian cells in the absence of genes encoding the DENV-2 E and prM glycoproteins. In this study, no contaminating E (gp60/55) or prM (gp20) glycoproteins were detected in immunoblot assays using high (200 ng) concentrations of the purified DENV-2 NS1 glycoproteins of the DENV-2 16681, NG-C or NSx strains with MAbs specific for each of these DENV glycoproteins (**Figure S1**).

### Immunogenicity and antigenicity of NS1 glycoproteins of different DENV-2 strains, and their ability to generate DENV-2 E glycoprotein cross-reactive PAbs

The ability of normal out-bred mice to generate PAbs against the native hexameric e/sNS1 glycoproteins of the DENV-2 16681, NG-C or NSx strains, that cross-reacted with the virion-associated E or prM glycoproteins was assessed. In this study, out-bred mice repeatedly immunized with the NS1 glycoproteins of either the DENV-2 16681, NG-C or NSx strains all generated high PAb titres against the NS1 glycoprotein of the NG-C strain (mean reciprocal log_10_t_50_ 4.50, 4.33 and 4.13 respectively) (**Table 1**). These PAbs, however, only weakly reacted with the NS1 glycoprotein of the DENV-2 NSx strain (mean reciprocal log_10_t_50_ 2.82 (47.9-fold reduction), 2.68 (44.7-fold reduction) and 2.81 (20.9-fold reduction), respectively. The NS1 glycoproteins of the DENV-2 16681, NG-C and NSx strains were, therefore, similarly immunogenic, but that of the NSx strain was very weakly antigenic in these ELISAs. The PAbs from each of the three mouse groups showed similar cross-reactive titres against purified DENV-2 virions of the NSx strain (mean reciprocal log_10_t_50_ 2.85, 2.78 and 2.65 respectively). Importantly, the PAbs generated against the NS1 glycoproteins of the DENV-2 16681 and NG-C strains showed higher mean ELISA titers against the virions, than the NS1 glycoprotein, of the DENV-2 NSx strain strongly suggesting that they were likely to generate AER of the DENV-2 NSx strain. Whilst PAbs generated in mice to live DENV-2 infections showed higher titers against the DENV-2 E glycoprotein, they also more weakly reacted with the NS1 glycoprotein of the DENV-2 NSx strain than the 16681 or NG-C strains.

**Table 1:** Mouse PAb and MAb reactions against DENV-2 virions and NS1 glycoproteins.

		50% end-point (1/log_10_t_50_) ELISA titer (s.d.)^c^			
		DENV-2 NS1 gp		DENV-2 Virions	
PAb/MAb^a^	Immunogen^b^	NG-C	NSx	NSx	Ovalbumin
Mouse PAbs	DENV-2 (16681) NS1 gp	4.50 (0.41)	2.82 (0.22)	2.85 (0.27)	1.76 (0.16)
Mouse PAbs	DENV-2 (NGC) NS1 gp	4.33 (0.41)	2.68 (0.25)	2.78 (0.29)	1.84 (0.16)
Mouse PAbs	DENV-2 (NSx) NS1 gp	4.13 (0.39)	2.81 (0.22)	2.65 (0.27)	1.71 (0.18)
Mouse PAbs	Ovalbumin	0.87 (0.07)	0.80 (0.06)	1.11 (0.12)	4.66 (0.24)
Mouse PAbs	DENV-2 (NGC) infections	3.38 (0.11)	2.15 (0.12)	5.25 (0.25)	1.21 (0.12)
MAb 2A5.1	DENV-2 (PR159) NS1 gp	5.86 (0.03)	5.81 (0.04)	0.60 (0.02)	0.41 (0.02)
MAb 2C5.1	DENV-2 (PR159) E gp	2.32 (0.02)	2.13 (0.02)	5.51 (0.03)	0.17 (0.01)
MAb 2A4.1	DENV-2 (PR159) prM gp	2.05 (0.02)	1.87 (0.01)	4.36 (0.04)	0.18 (0.01)

a Mouse monoclonal antibodies (MAbs) specific for the NS1 (2A5.1), E (2C5.1) or prM (2A4.1) glycoproteins or polyclonal antibodies (PAbs) generated against DENV-2 infections or immuno-affinity purified DENV-2 NS1 glycoproteins, and which were collected immediately prior to challenge with either the DENV-2 NG-C or NSx strains.

b Immunogen as either the immuno-affinity purified NS1 glycoprotein (NS1 gp) of the DENV-2 16681, NG-C or NSx strains, ovalbumin (control protein) or repeated live DENV-2 (NG-C strain) infections.

c The mean reciprocal log_10_ 50% end-point ELISA titer (1/log_10_t_50_) and standard deviation (s.d.) of pools of mouse PAbs or MAbs, against the immuno-affinity purified NS1 glycoproteins of the DENV-2 NG-C or NSx strains, ovalbumin (control protein) or purified DENV-2 (NSx strain) virions, when detected using peroxidase-labelled goat anti-mouse IgG (H&L) second antibodies. Mouse PAbs which had higher ELISA titers against the DENV-2 virions of the NSx strain than its NS1 glycoprotein are underlined.

**Figure 1:**
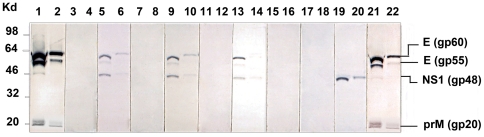
Immunoblot reactions of PAbs and MAbs against DENV-2 proteins. Approximately 1000 ng (odd numbered lanes) and 250 ng (even numbered lanes) concentrations of purified DENV-2 (NSx strain) virions, obtained from infected C6/36 supernatants, were heated at 100°C for 3 min and subjected to 9% (wt/vol) non-reduced SDS-PAGE and immuno-blotting. These strips were then reacted with either 1/200 dilutions of mouse PAbs generated against repeated infections with DENV-2 (NG-C strain) (lanes 1 and 2) or the pre-immunization (pre-i) and post-immunization (post-i) sera pooled from mice that had been generated against the purified NS1 glycoproteins of the DENV-2 16681 (pre-i: lanes 3 and 4; post-i: lanes 5 and 6), NG-C (pre-i: lanes 7 and 8; post-i: lanes 9 and 10) or NSx (pre-i: lanes 11 and 12; post-i: lanes 13 and 14) strains or ovalbumin (pre-i: lanes 15 and 16; post-i: lanes 17 and 18) or 1 µg/ml of IgG2a subclass MAbs specific for the DENV-2 NS1 (MAb 2A5.1) (lanes 19 and 20) the DENV E and prM glycoproteins (MAbs 2C5.1 and 2A4.1) (lanes 21 and 22) (**Table 1**). The bound MAbs were then detected using a peroxidase-labelled goat anti-mouse IgG2a subclass-specific secondary PAbs and CND substrate. The locations of standard kilodalton (kD) molecular weight markers and the DENV-2 E (gp60 and 55), NS1 (gp48) and prM (gp20) glycoproteins are shown.

The cross-reactions of the PAbs generated against the purified NS1 glycoproteins of the DENV-2 16681, NG-C and NSx with the virion-associated E or prM glycoproteins were further tested using immunoblot assays. For this study, purified DENV-2 (NSx strain) virions obtained from infected C6/36 mosquito cells were used to more easily distinguish between the E (gp60 and gp55) and NS1 (gp48) glycoprotein bands (**Figure 1**). In addition, the ability to detect PAbs of the IgG2a subclass was assessed. In this study, the PAbs generated against the purified e/sNS1 glycoproteins of each DENV-2 strain all generated detectable PAbs of the IgG2a subclass that reacted with the NS1 glycoprotein (gp48), and cross-reacted with the E glycoprotein (gp60 and gp55) of the DENV-2 NSx strains, but not with its prM glycoprotein (gp20) (**Figure 1**). All of these PAbs had a low plaque-reduction neutralisation titer (PRNT) of 1/8 against both the DENV-2 NG-C and NSx strains, suggesting that they may also generate DENV-2 AER. In contrast, PAbs generated in mice against repeated live DENV-2 infections (anti-E, prM and NS1 glycoprotein PAbs) or anti-DENV E and prM glycoprotein-specific MAbs, 2C5.1 and 2A4.1, strongly reacted with the DENV-2 E and prM glycoproteins but less strongly with the NS1 glycoprotein, while MAb 2A5.1 showed a strong anti-DENV-2 NS1 glycoprotein-specific reaction. All of their pre-immunisation PAb sera, as well as those generated against ovalbumin (control glycoprotein), however, failed to cross-react with any DENV-2 glycoproteins (**Table 1** **,** **Figure 1**).

### Ability of PAbs raised against the NS1 glycoproteins of DENV-2 strains to generate antibody-enhanced disease (AED) in mice

**Figure 2:**
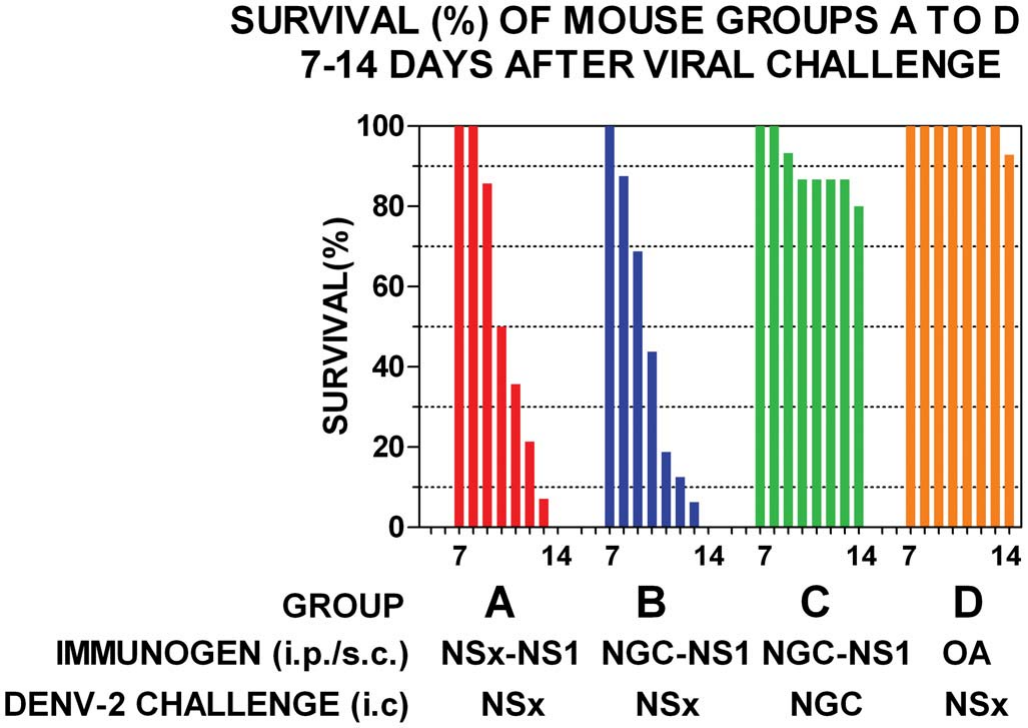
Ability of DENV-2 NS1 glycoproteins to generate DENV-2 AED. Groups of 14-16 out-bred mice were repeatedly immunized with the DENV-2 NS1 glycoproteins of either the NSx (NSx-NS1) (Group A), NG-C (NGC-NS1) (Group B and C) strains, or ovalbumin (OA) (Group D) (immunogens) by the combined i-p./s-c. route, and challenged by the intra-cerebral route with a low (<0.5 LD_50_) dose of either the DENV-2 NSx (Group A, B and D) or NG-C strain (Group C). The survival (%) for each group is shown from day 7 to 14 after DENV-2 challenge by colored bars and Kaplan-Meier survival curves were compared to obtain statistical values for groups A versus B (χ2: 0.06; p = 0.81), A versus C (χ2 = 20.91; p<0.0001), A versus D (χ2 = 20.80; p<0.0001) and B versus C (χ2 = 20.80; p<0.0001).

**Figure 3:**
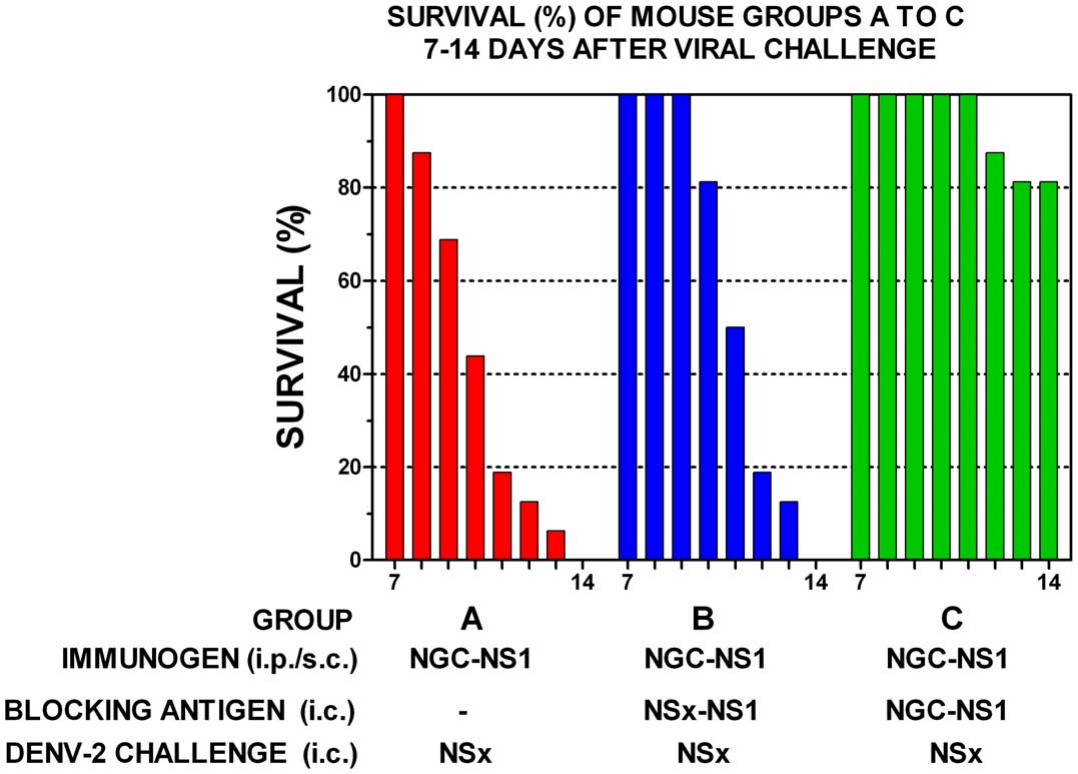
Ability of DENV-2 NS1 glycoproteins to block the DENV-2 AED. Groups of 14-16 out-bred mice were repeated immunized with the immuno-affinity DENV-2 NS1 glycoprotein of the NG-C strain (NGC-NS1glycoprotein immunogen) by the combined i-p./s-c. route, and either pre-treated intra-cerebrally with protein-free RPMI medium (A), or pre-treated intra-cerebrally with 500 µg of the purified NS1 glycoproteins of either the DENV-2 NSx strain (NSx-NS1) (B) or the NG-C strain (NGC-NS1) (C), immediately prior to and at the same intra-cerebral site as the subsequent challenge with a low dose (<0.5 LD_50_) of the DENV-2 NSx strain. The survival (%) for each group is shown from day 7 to 14 after challenge by colored bars and Kaplan-Meier survival curves were compared to obtain values for groups A versus B (χ2 = 7.10; p <0.008), A versus C (χ2 = 27.46; p<0.0001) and B versus C (χ2 = 23.84; p<0.0001).

Groups of out-bred mice immunized with either the NS1 glycoproteins of the DENV-2 NG-C or NSx strains and challenged with a low dose (<0.5 LD_50_) of the live DENV-2 NSx strain all showed symptoms of severe respiratory distress and hind-leg paralysis on, or before, day 14 after infection (**Figures 2A and 2B**). Only 3/15 (20%) of the mice immunized with the NS1 glycoprotein of the DENV-2 NG-C strain and challenged with a low dose (<1 LD_50_) of live DENV-2 NG-C strain, however, showed symptoms of severe respiratory distress and hind-leg paralysis on, or before, day 14 after challenge (**Figure 2C**), after which no further cases of morbidity occurred. In contrast, only 1/14 (7.1%) of the mice immunized with the control protein, ovalbumin (OA) and challenged with a low dose of the live DENV-2 NSx strain displayed hind-leg paralysis on, or before, day 14 after challenge (**Figure 2D**), but this animal did not display severe respiratory distress, and none of the other 13/14 animals showed any signs of morbidity. These results using the control mice therefore confirmed that the deaths that occurred in the other mouse groups (2A, 2B or 2C) were not caused by another contaminating infectious agent or brain material. These results were strongly supported by significant Kaplan-Meier survival curve statistics (Figure 2: group A versus C: χ2 20.91, p<0.0001; group B versus C: χ2 20.84, p<0.0001; group A versus D (control): χ2 30.04, p<0.0001; group B versus D (control): χ2 29.87, p<0.0001). Thus, the immunization of out-bred mice with the native NS1 glycoproteins of either the DENV-2 NG-C or NSx strains generated PAbs that caused dramatic and statistically significant, DENV-2 AED when they were challenged with the DENV-2 NSx strain, but not the DENV-2 NG-C strain. These results indicated that the DENV-2 AED was generated only when the mice were immunized with DENV-2 NS1 glycoproteins and challenged with the DENV-2 NSx strain, because of their relatively stronger PAb reactions against its E glycoprotein, and weaker (44.7-fold and 20.9-fold reduced) antigenicity of its NS1 glycoprotein (**Table 1**). To confirm this hypothesis, the ability of the NS1 glycoproteins of the DENV-2 NG-C or NSx strains to block the DENV-2 AED was tested. In this study, three groups (A, B and C) of 14-16 three-week old out-bred mice were all immunized with the immuno-affinity purified multi-meric e/sNS1 glycoprotein of the DENV-2 NG-C strain, and again boosted two weeks later with the same antigen dose in PBS. One week later (i.e. at six-weeks old), 250 µl of sterile protein-free RPMI medium (group A: controls) was administered intra-cerebrally to each mouse in group A immediately prior to, and at the same site as, the challenge dose containing <0.5 LD_50_ of the DENV-2 NSx strain. Each mouse in the other groups received 500 µg of the purified DENV-2 e/sNS1 glycoprotein of either the NSx (group B) or NGC (group C) strains by the intra-cerebral route immediately prior to, and at the same site as, challenge with <0.5 LD_50_ of the DENV-2 NSx strain. In this study, all of the mice immunized with the DENV-2 NS1 glycoprotein, that received protein-free RPMI medium prior to challenge with the DENV-2 NSx strain (group A: controls) developed severe respiratory distress and hind-leg paralysis on, or before, day 14 after infection (**Figure 3A**). The immunized mice that were pre-injected with the NS1 glycoprotein of the NSx strain immediately prior to challenge with the DENV-2 NSx strain (group B) showed a delayed onset of severe morbidity, but they all subsequently succumbed to severe respiratory distress and hind-leg paralysis on, or before, day 14 after infection (**Figure 3B**). Despite these findings, a statistically significant difference was obtained between these two groups (Figure 3: group A versus B: χ^2^ 7.10, p<0.008). In contrast, only 3/15 (20%) of the immunized mice that were pre-injected with the NS1 glycoprotein of the DENV-2 NG-C strain, immediately prior to challenge with the DENV-2 NSx strain (group C), developed severe respiratory distress and hind-leg paralysis on, or before, day 14 after infection. The NS1 glycoprotein of the DENV-2 NGC strain therefore more strongly blocked the DENV-2 AED, with 12/15 (80.0%) of these mice surviving (i.e. showing no severe respiratory distress and/or hind-leg paralysis) on day 14 after challenge (**Figure 3C**) (Figure 3: group B versus C: χ^2^ 23.84, p<0.0001). There was also no significant difference between the blocking of AED by the NS1 glycoprotein of NG-C (**Figure 3C**) and the DENV-2 non-AED mice immunised with ovalbumin (control glycoprotein) before viral challenge (**Figure 2D**) (Figure 3 group C versus Figure 2 group D: χ^2^ 1.07, p = 0.302). Thus, the treatment of the mice with the NS1 glycoprotein of the DENV-2 NG-C strain significantly blocked all evidence of DENV-2 AER/AED, while the NS1 glycoprotein of the DENV-2 NSx strain failed to prevent AED, probably due to its much weaker antigenicity (**Table 1**) (Figure 3: group B versus Figure 2 group D: χ^2^ 29.70, p<0.0001).

Brain homogenates were prepared from the mice on day 14 after challenge to confirm that the AED was caused by DENV-2 AER. Since the high lipid and protein concentrations in these homogenates affected DENV-2 plaque-formation, initial dilutions were started at 1/10 of the 10% (wt/vol) brain homogenates (i.e. 1/100 dilution/gram of brain homogenate). In these assays, the DENV-2 AED mice tested (n = 6) showed consistent DENV-2 AER with an average DENV-2 NSx strain titer of 8.5×10^7^ (standard deviation 2.6×10^6^) plaque-forming units/gram of brain homogenate, while no plaques could be detected in brain homogenates of the non-AER/AED animals at the starting dilution of 1/400. The average DENV-2 NSx strain AER was therefore at least 90,000-fold, but was likely to be higher since DENV-2 E glycoproteins could not detected in the brains of the DENV-2 non-AER/AED mice by immuno-histology (see later). A dramatic AER of the DENV-2 NSx strain which resulted in DENV-2 AED was, therefore, confirmed in these animals.

DENV-2 was also isolated from lung, spleen and liver homogenates from each of four DENV AER/AED mice that died on day 8-9 after challenge using C6/36 cells. These results, therefore, confirmed that the DENV-2 NSx strain had spread to infect these peripheral organs.

### Histological studies on organs from the DENV-2 AER/AED mice and non-AER/AED mice

**Figure 4:**
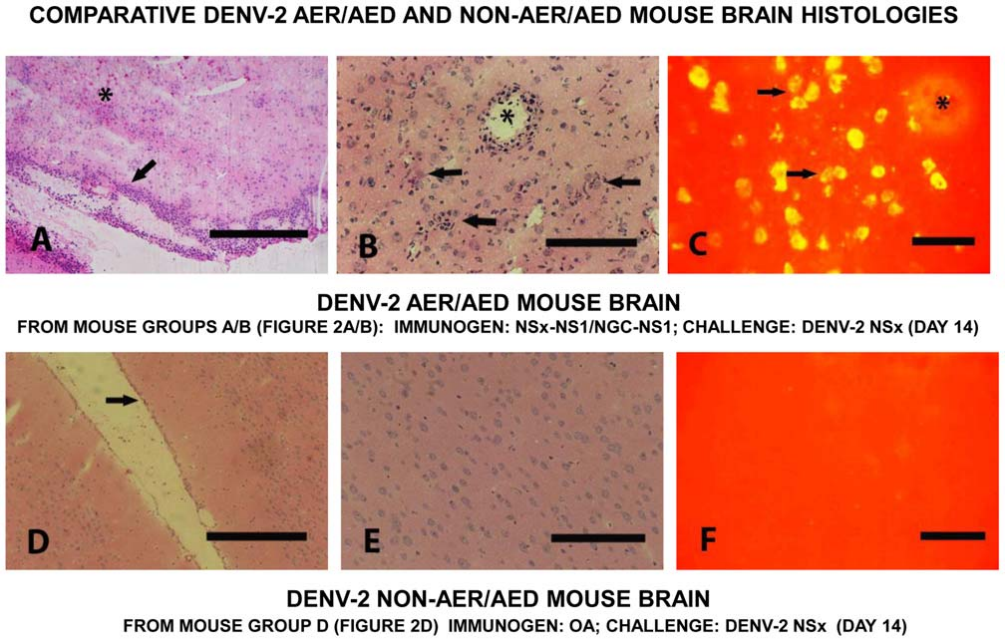
Comparative DENV-2 AER/AED and non-AER/AED mouse brain histologies. Representative DENV-2 AER/AED mouse brain sections (A, B and C) from group A/B mice (Figure 2A/B) and DENV-2 non-AER/AED mouse brain sections (D, E and F) from group D mice (Figure 2D), immunized with either the NS1 glycoprotein of the DENV-2 NSx strain or ovalbumin by the combined i-p./s-c. routes, respectively, prior to challenged with the DENV-2 NSx strain by the i-c. route, collected on day 14 after challenge. Cryostat-cut sections were either stained with standard hematoxylin and eosin (H&E) (A, B, D and E) or tested in immuno-fluorescence antibody (IFA) assays using human PAbs reactive against the DENV E glycoprotein and FITC-labelled secondary PAbs. The DENV-2 AER/AED mouse meninges showed extensive mononuclear cell infiltration (arrowed) with microglial nodule formation and many eosinophilic-staining dead neurones (asterixed) (100x: 100 µm bar) in their brain parenchyma (A). The DENV-2 AER/AED mouse microglial nodules were located throughout their brain parenchyma (arrowed) and blood vessels showed perivascular cuffing with mononuclear cells (asterixed) (200x: 50 µm bar) (B). DENV-2 E glycoproteins were present in the microglial cells, including those which formed nodules (arrowed) throughout their brain parenchyma, but not in the mononuclear cells in the perivascular infiltrate (asterixed) (400x: 20 µm bar) (C). In contrast, the DENV-2 non-AER/AED mice showed normal meninges (arrowed) (100x: 100 µm bar) (D) and no pathological changes in their brain parenchyma (200x: 50 µm bar) (E) and no DENV-2 E glycoproteins were present in these cells (400x: 20 µm bar) (F).

Normal histological and immuno-histological analyses on various organs of the DENV-2 AED mice (groups A and B from Figure 2A and 2B) and DENV-2 non-AER/AED mice (group D from Figure 2D), collected on day 14 after infection, were performed to i) contrast the pathologies observed in these organs, ii) identify the DENV-2 antigen-positive cell-types in these organs, and iii) compare these results with those reported in DHF/DSS patients' organs. In this study, the DENV-2 AED mice (groups A and B) all showed severe meningitis and displayed dramatic mononuclear cell infiltration, predominantly of lymphocytes and plasma cells, over a background of fibrinoid material (**Figure 4A**). The encephalitis was characterized by the presence of eosinophilic-staining dead neurones and lymphocytic infiltration of the brain parenchyma, with the formation of microglial cell nodules around necrotic neurons (**Figures 4A and 4B**). Perivascular lymphocytic cuffing, composed mainly of lymphocytes and plasma cells with a thickness ranging from 2-5 cell layers, was evident in the brain hemispheres (**Figures 4A and 4B**). Interestingly, DENV-2 E glycoproteins were not identified in the mononuclear cells within either the meninges or the perivascular infiltrate in the brain parenchyma, but were identified in the phagocytic microglial cells, including those that formed nodules throughout the brain parenchyma (**Figure 4C**). These histopathological changes were predominantly observed in the grey matter, consistent with its higher density of neurons. The microglial nodule formation and apoptotic neurones observed in the DENV-2 AER/AED mice also accorded with histological studies on encephalitis in humans or mice caused by neurotropic flaviviruses (e.g. West Nile [62] or Japanese encephalitis [63] viruses). In contrast, the DENV-2 non-AED mice (group D) showed normal meninges and brain parenchyma (**Figures 4D and 4E**), consistent with the inability to detect any DENV-2 E glycoproteins in any cells within these tissues (**Figure 4F**).

**Figure 5:**
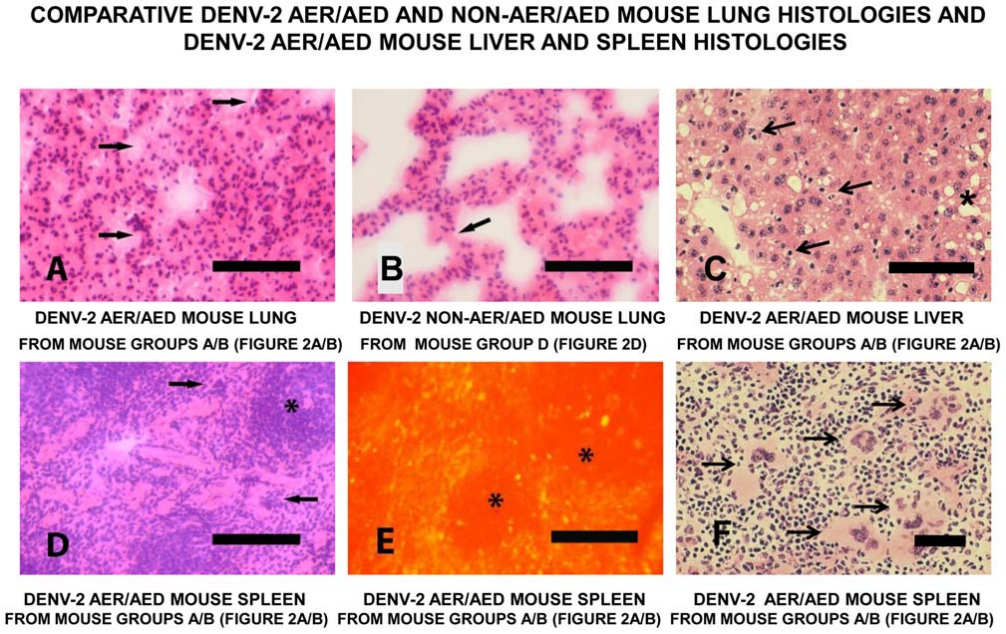
Comparative DENV-2 AER/AED and non-AER/AED mouse lung histologies and DENV-2 AER/AED mouse liver and spleen histologies. Representative DENV-2 AER/AED mouse lung, liver and spleen sections (A, C, D, E and F) from group A/B mice (Figure 2A/B) and DENV-2 non-AER/AED mouse lung sections (B) from group D mice (Figure 2D), immunized by the combined i-p/s-c. route with either the NS1 glycoprotein if the DENV-2 NSx strain or ovalbumin, respectively, and challenged with the DENV-2 NSx strain by the i-c. route, collected on day 14 after challenge. Cryostat-cut sections were either stained with H & E (A, B, C, D and F) or tested in immuno-fluorescence antibody (IFA) assays using human PAbs reactive against the DENV E glycoprotein and FITC-labelled second PAbs (E). The DENV-2 AER/AED mouse lung showed extensive edema, mononuclear cell infiltration with dramatic alveolar wall-thickening, protein-rich fluid-filled alveolar spaces (arrowed) and hyaline membrane formation (200x: 50 µm bar) (A). In contrast, the DENV-2 non-AER/AED mouse lung showed slight oedema, with some alveolar walls still one cell thick (arrowed) and no hyaline membrane formation (200x: 50 µm bar) (B). The DENV-2 AER/AED mouse liver showed some mononuclear cell infiltration, macro-steatosis (asterixed) and micro-steatosis, with many cells displaying apoptosis (pyknotic nuclei) (some arrowed) (200x: 50 µm bar) (C). The DENV-2 AER/AED mouse spleen contained numerous megakaryocytes, with characteristic multi-lobed nuclei, in the red pulp (arrowed) (D) but not the white pulp (asterixed) (200x: 50 µm bar), with some arrowed in a higher magnification photomicrograph (400x: 25 µm bar) (F), and DENV-2 E glycoprotein was found in the cells throughout the red pulp, but not the white pulp (asterixed) (200x: 50 µm bar) (E).

The lungs of the DENV-2 AER/AED mice showed dramatic edema and a severe state of acute respiratory distress syndrome (ARDS) displayed by diffuse alveolar damage (DAD), with the extensive thickening of the alveolar walls by the infiltration of macrophages and lymphocytes and occasional hyperplasia of alveolar type II pneumocytes (**Figure 5A**). In addition, there was copious intra-alveolar proteinaceous secretion with the occasional formation of hyaline membrane-covered alveolar walls. DENV-2 E glycoproteins were present in their alveolar macrophages, which had characteristic morphologies (data not shown). These findings were therefore typical of life-threatening ARDS, thereby accounting for the severe symptoms of respiratory distress displayed by these animals. In contrast, the DENV-2 non-AER/AED mice showed a slight degree of edema in their lungs, but many alveolar walls were still one cell thick, there was no hyaline membrane formation (**Figure 5B**), and no DENV-2 E glycoproteins were detected in their alveolar macrophages. These results therefore accorded with the histo-pathological descriptions of lung pathology observed in fatal DHF/DSS cases, in which oxygen-exchange was severely inhibited [26], and as was observed in histological photomicrographs obtained from a fatal DHF/DSS case [59]. The livers of the DENV-2 AER/AED mice displayed extensive necrosis and micro- and macro-steatosis, with many cells diplaying pyknosis (apoptosis) (**Figure 5C**). DENV-2 E glycoproteins were identified in many Kuppfer cells with their characteristic morphologies [61], and hepatocytes using immuno-fluorescent microscopy (data not shown). The DENV-2 AER/AED mice, therefore, demonstrated severe liver disease, while the DENV-2 non-AER/AED control mice displayed normal liver histologies. These results therefore accord with those described in fatal cases of ‘severe dengue’ disease in humans [5], [26], [33], [34]. High numbers of megakaryocyte (platelet precursor) cells, with their characteristic multi-lobed nuclei, had infiltrated the spleens of the DENV-2 AER/AED mice, and DENV-2 E glycoproteins were identified in many of the macrophages located throughout the red pulp, but in only relatively low numbers of cells in the white pulp (**Figures 5D, 5E and 5F**). The DENV-2 non-AER/AED mice also showed some infiltration of megakaryocytes in their spleens (data not shown), but DENV-2 E glycoproteins were not detected in their spleens. These results, together with the ability to isolate DENV-2 from lung, spleen and liver homogenates from the DENV AER/AED mice on day 8–9 after challenge, therefore, confirmed that the DENV-2 NSx strain had spread to infect the peripheral organs, probably when the blood-brain barrier was breached during DENV-2 challenge, and when bleeding was observed at these injection sites.

## Discussion

The main findings from this study were: 1) that PAbs generated against the DENV-2 NS1 glycoprotein could cross-react with the E glycoprotein and generate the highest DENV (≥90,000-fold) AER titers so far reported *in vivo* using undiluted PAbs with a wild-type DENV-2 strain, 2) that this dramatic DENV-2 AER/AED could be blocked using a more antigenic NS1 glycoprotein of another DENV-2 strain, 3) that DENV-2 was confirmed to have spread to the peripheral organs by isolation of the virus after intra-cerebral challenge, and that intra-cerebral challenge was a suitable route for testing the ability of PAbs to provide either DENV-2 protection or AED in both the CNS and peripheral organs, 4) that this was the first report in which severe, life-threatening DENV acute respiratory distress syndrome (ARDS) was generated in an animal model, 5) that this was also the first study to observe increased megakaryocyte (platelet precursor) cell numbers resulting from DENV-2 AER/AED in an animal model, 6) that the severe pathological findings in lung (ARDS), brain (encephalitis), liver (necrosis and apoptosis (pyknosis), with macro- and micro-steatosis) and spleen samples with DENV-2 antigen-positive tissue macrophages from the DENV-2 AER/AED animals accorded with those found in fatal human ‘severe dengue’ cases, and 7) that candidate DENV NS1 glycoprotein-based vaccines may thus be hazardous, particularly when used against DENV strains that possess less antigenic NS1 glycoproteins.

These PAbs did not increase the replication of the prototype DENV-2 NG-C strain, probably due to the stronger antigenicity of its NS1 glycoprotein, which was supported by the ability of its NS1 glycoprotein to block the DENV-2 AER/AED caused by the NSx strain. The NS1 glycoprotein of the DENV-2 NSx strain was, therefore, chosen as a natural low passage DENV-2 isolate to demonstrate the proof of principle that PAbs raised against the NS1 glycoprotein candidate vaccine could generate DENV-2 AER under physiological conditions (i.e. undiluted PAbs in the presence of complement and auto-antigens). Interestingly, a MAb generated against the DENV prM glycoprotein that also cross-reacted with proteins on mammalian cells generated DENV AER in the absence of FcRs *in vitro* [65]. Thus, while the DENV-2 AER generated here was likely to be FcγR-dependent, the dual specificities of these PAbs, against both the DENV-2 E glycoprotein and host cell-surface auto-antigens [23]–[25] may also occur through a FcγR-independent mechanism. We opted to assess this possibility using panels of MAbs (e.g. MAb 1G5.4-A1-C3), rather than these mouse PAbs which contain antibodies of different IgG subclasses, since the double-cleavage reactions required to obtain F(ab')_2_ fragments of IgG1 and other IgG subclasses [66], [67] was likely to result in the disruption of antigenic binding. This study demonstrates the first evidence that PAbs raised against the DENV NS1 glycoprotein could generate a dramatic AER of a DENV-2 strain in out-bred mice *in vivo*, with lethal multi-organ disease similar to that observed in the most severe and lethal DSS cases. These results therefore raise further concerns, in addition to the ability to generate auto-immune disease [24], [25], over the safety of any DENV NS1-based candidate vaccines.

The challenge route used for the DENV-2 NSx AER/AED experiments was by intra-cerebral inoculation, as has been used as for testing DENV active and passive protection experiments [46]. This model has therefore been extensively used to evaluate the protective capacity of neutralizing PAbs and MAbs generated against the DENV E and prM glycoproteins, as well as non-neutralising PAbs and MAbs generated against the DENV NS1 and C proteins [47]–[52]. We have also generated this DENV-2 AER/AED in CD1 Swiss (out-bred) and BALB/c (inbred) mouse strains, thereby suggesting that any mouse strain may be used for this model (data not shown). In a previous study, we showed that mice challenged with sub-lethal doses of DENV-2 by the intra-cerebral route, generated peak titers 8 days later, but which became undetectable on day 10 [9]. In contrast, lethal DENV-2 AER reached maximum titers on 9 days after challenge, and remained the same until their deaths on day 12–14 after challenge [9]. As such, the control mice generated much lower DENV-2 titers and showed no disease symptoms, probably due to DENV-2 clearance by the rising titers of protective PAbs. This was consistent with our inability to detect any DENV-2 virus, antigens or pathology in the brains of the non-AER/AED mice on day 14 after challenge in this study. Microglial cells are the principal resident macrophages in the CNS, and which express all four classes of mouse FcγRs [68]. Their FcγRI-expression was greatly increased by IFN-γ [69] and they were also activated by antigen-IgG complexes binding to their Fc-γRIs (IgG2a only) and Fc-γRIIIs [68], which resulted in MIP-1α-release and neuronal apoptosis, and which has been implicated in a wide range of neurological diseases [70]. Of particular concern is that DENV encephalitis has increasingly been reported in both Asia and South America [71], was the principal cause of encephalitis in one DENV-endemic area [42], 7% in studies conducted in Jamaica and Indonesia [43], [44], and 4.6% in a study conducted in Viet Nam, where Japanese encephalitis virus was prevalent [45]. DENV encephalitis has been associated with a poor patient prognosis [41], and has been added as a symptom of ‘severe dengue’ by the TDR/WHO steering committee [3]. Our DENV-2 AER/AED model is, therefore, likely to be valuable in testing potential therapies for these patients.

Despite using the unnatural intra-cerebral challenge route, the DENV-2 was disseminated to the peripheral organs of the DENV-2 AER/AED mice at the time of DENV-2 challenge when the blood-brain barrier was breached, and bleeding was observed at these injection sites. This was confirmed by isolation of DENV-2 by cell culture from the lungs, spleens and livers of the DENV-2 AER/AED mice on day 8–9 after challenge. These results, therefore, support those previously found in liver samples of mice after DENV challenge doses by the intra-cerebral route [53], [54]. This challenge route, therefore, yielded very clear hind-leg paralysis and life-threatening ARDS end-points for the DENV AER/AED and blocking studies, which will be very useful for passive protection studies using both PAbs and MAbs (see below).

We previously showed that some MAbs of the non-complement-fixing IgG1 subclass (e.g. MAb 1G5.3) that were generated against the DENV-2 NS1 glycoprotein identified common epitopes on the DENV E glycoproteins, weakly neutralised them [23], and also generated DENV-2 AED [9]. Affinity purified IgG obtained from out-bred mice immunized with the DENV-2 NS1 glycoprotein have also been used to generate DENV-2 AER/AED in naïve mice after challenge with the DENV-2 NSx strain (Falconar, manuscript in prep). MAbs of the IgG1, IgG2b and IgG2a subclasses, some of which fixed serum complement (e.g. MAb 1G5.4-A1-C3: IgG2b subclass), and also defined common epitopes on the DENV E and NS1 glycoproteins [23], have been tested for their abilities to generate DENV AER/AED resulting in similar multi-organ pathologies in mice (Falconar, manuscript in prep).

While there have been differences reported in the ability of DENVs to infect cells of the non-monocyte/macrophage lineages (e.g. lymphocytes, hepatocytes, endothelial cells and megakaryocytes) [5], [26]–[34], [72], [73], Fc receptor bearing monocytes and tissue macrophages are universally considered to be the principal target cells for DENV replication. This was confirmed in the DENV-2 AER/AED mice by finding that, with the exception of hepatocytes, DENV-2 antigens were only found in tissue macrophages possessing their characteristic morphologies, in each of the organs studied (lungs, livers, spleens and brains). In our study, we also observed that much higher percentages of the macrophages present in the splenic red pulp and liver, rather than the lungs, contained DENV-2 E glycoproteins, consistent with the spleen, as well as the liver, being a principal site for DENV replication [27], together with high megakaryocte numbers in the spleen. This was, therefore, the first observation of increased megakaryocyte numbers in animals infected with DENV, as has been a frequent observation in histological studies on DHF/DSS patient autopsies [5], [26]. Young megakaryocytes were, however, reported to be present in both the bone marrow and peripheral organs of DHF/DSS patients [5], [74], but in other reports these increased numbers of megakaryocytes displayed vacuolation or disintegration, which subsequently resulted in bone marrow suppression [72], [73]. Since the megakaryocytes located in the splenic red pulp of the DENV-2 AER/AED mice were morphologically mature, further studies are required to account for these different observations.

Interestingly, pulmonary congestion with liver steatosis was observed in BALB/c after the administration of high doses of a low-passage DENV-2 strain by the peripheral route [75]. These symptoms were, therefore, similar to those observed in our study, but those mice only transiently displayed the severe lung congestion before it was resolved, and no mortalities occurred. In contrast, the ARDS was sufficiently severe and prolonged (studied on day 14 after DENV challenge) in our DENV-2 AER/AED mice, that it *per se* could cause death, and probably also contributed to the severe liver necrosis with macro- and micro-steatosis observed in these animals, since multi-organ or systemic pathology due to hypoxia and metabolic acidosis are common complications of ARDS [76], [77]. Importantly, in several reports ARDS was the main cause of mortality in DSS patients either alone or through its cause of, or contribution to, multi-organ failure and DIC, and this often caused death even when these patients received early fluid replacement [36]–[39], and after their plasma leakage was resolved [26]. The previous results [75], together with finding of severe pathology in the peripheral organs of the DENV-2 AER/AED mice, from which DENV-2 was isolated, strongly suggest that our DENV-2 AER/AED model may also be used when the DENV-2 NSx strain is delivered by the intra-peritoneal challenge route. This is therefore the first report demonstrating severe life-threatening DENV-2 induced ARDS in an animal model. Further studies are also needed however to identify the role of auto-antibody reactions [24], [25], complement, and cytokines/chemokines secreted from different macrophage populations and T-cells in the different organ pathologies observed in these mice.

AG129 mice, deficient in IFN-α, β and γ receptors, generated antibodies of IgG1, but not the IgG2a, subclasses [78], were also not protected by MAbs of the IgG2a subclass (e.g. MAb 4G2: CF titer: 1/16 [56]) when administered at 50 µg concentrations that would solidly protect normal mice [79]. AG129 mice also did not show pathological symptoms in their lungs when infected with different DENV-2 strains [80], [81], or when low concentrations of PAbs, generated against live DENV-1 infections in AG129 mice, were passively administered (100 µl of serum/mouse) to naïve AG129 mice before challenge with DENV-2 [82]. These results were in stark contrast to those observed in our normal mice, and in fatal DSS cases [5], [26], probably due to the failure of AG129 mice to activate their macrophages and other ADCCs with IFN-γ [83]. This would be consistent with the higher levels of IFN-γ and macrophage-activation reported in DHF/DSS patients [84], [85].

IFN-γ from splenic NK cells, together with rising antibody titers, resulted in rapidly reduced DENV-2 replication in A/J mouse spleens [86]. Similarly, peak DENV-2 titers occurred in the spleens of AG129 mice, before being reduced soon after DENV challenge [81], [87], [88]. Thus, our ability to isolate the DENV-2 from lung, spleen and liver samples collected on day 8-9 after challenge suggested that clearance from these organs was delayed due to its AER.

Since the DENV E and NS1 glycoproteins appeared to co-evolve antigenically [89], variations in their antigenicities and therefore their potentials to generate AER/AED are likely to occur through mutations or genetic recombination events. Recombination has been identified in the genes encoding the E and NS1 glycoproteins of a number of DENV strains of the same, as well as different genotypes [90]–[92], and in one study occurred between strains of the DENV-2 American (weakly pathogenic), Asian/American (highly pathogenic) and Cosmopolitan (pathogenic) genotypes [92]. The NS1 glycoprotein of the DENV-2 NSx strain therefore appeared to have a reduced antigenicity due to either multiple amino acid substitutions, or possibly by a major recombination event between heterologous DENV-2 genotypes or a different DENV serotype. These possibilities are being investigated using DENV-2 NSx cDNA sequence determination and AER/AED studies using panels of MAbs generated against the DENV NS1 glycoprotein, which defined single or multiple epitopes on DENV NS1 and E glycoproteins of different DENV strains [23]–[25].

Most importantly, blocking DENV AER/AED to prevent the ARDS and multiple organ dysfunction syndrome (MODS), would be particularly useful for DSS patients.

## Supporting Information

Figure S1 **Immunoblot reactions of immuno-affinity purified NS1 glycoproteins.** High (960 ng) concentrations of purified DENV-2 (NSx strain) virions (lanes 1 and 5) and high (200 ng) concentrations of the purified e/sNS1 glycoproteins of the 16681 (lanes 2 and 6), NG-C (lanes 3 and 7) and NSx (lanes 4 and 8) were heated at 100°C for 3 min and subjected to 8% (wt/vol) non-reduced SDS-PAGE and immuno-blotting. These strips were then reacted with 1 µg/ml concentrations of MAbs specific for either the DENV NS1 (MAb 2A5.1) glycoprotein (lanes 1 to 4) or the E (MAb 2C5.1) and prM (MAb 2A4.1) (lanes 5 to 8) glycoproteins. The bound MAbs were then detected using peroxidise-labelled anti-mouse IgG2a subclass-specific secondary PAbs and CND substrate. The location of the standard kD molecular weight markers and the DENV-2 E (gp60/55), e/sNS1 (gp48) and prM (gp20) glycoproteins are shown.
(TIF)

## References

[1] Gubler DJ (2006). Dengue/dengue haemorrhagic fever: history and current status.. Novartis Found Symp.

[2] World Health Organization (WHO) (1997). Dengue haemorrhagic fever: diagnosis, treatment, and control. 2^nd^ ed..

[3] World Health Organization (WHO) (2009). Dengue haemorrhagic fever: diagnosis, treatment, and control..

[4] Halstead SB (2003). Neutralization and antibody-dependent enhancement of dengue viruses.. Adv Virus Res.

[5] Bhamarapravati N, Gubler DJ, Kuno G (1997). Pathology of dengue infections.. Dengue and dengue hemorrhagic fever.

[6] Pang T, Cardosa MJ, Guzman MG (2007). Of cascades and perfect storms: the immunopathogenesis of dengue haemorrhagic fever-dengue shock syndrome (DHF/DSS).. Immunol Cell Biol.

[7] Gould EA, Buckley A, Groegar BK, Cane PA, Doenhoff M (1987). Immune enhancement of yellow fever virus neurovirulence: studies of mechanisms involved.. J Gen Virol.

[8] Gould EA, Buckley A (1989). Antibody-dependent enhancement of yellow fever and Japanese encephalitis virus neurovirulence.. J Gen Virol.

[9] Falconar AKI, Pandalai SG (1999). The potential role of antigenic themes in dengue viral pathogenesis.. Recent research developments in virology.

[10] Kliks S, Nisalak A, Brandt WE, Wahl L, Burke DS (1989). Antibody dependent enhancement of dengue virus growth in human monocytes as a risk factor for dengue haemorrhagic fever.. Am J Trop Med Hyg.

[11] Chau TN, Quyen NT, Thuy TT, Tuan NM, Hoang DM (2008). Dengue in Vietnamese infants-results of infection-enhancement assays correlate with age-related disease epidemiology, and cellular immune responses correlate with disease severity.. J Infect Dis.

[12] Halstead SB, Shotwell H, Casals J (1973). Studies on the pathogenesis of dengue infection in monkeys. II. Clinical laboratory responses to heterologous infection.. J Infect Dis.

[13] Thein S, Aaskov J, Myint TT, Shwe TN, Saw TT (1993). Changes in levels of anti-dengue virus IgG subclasses in patients with disease of varying severity.. J Med Virol.

[14] Kontny U, Kurane I, Ennis FA (1988). Gamma interferon augments Fc gamma receptor-mediated dengue virus infection of human monocytic cells.. J Virol.

[15] Littaua R, Kurane I, Ennis FA (1990). Human IgG Fc receptor II mediates antibody-dependent enhancement of dengue virus infection.. J Immunol.

[16] Jefferis R, Lund J (2002). Interaction sites on human IgG-Fc for FcgammaR: current models.. Immunol Lett.

[17] Nimmerjahn F, Ravetch JV (2006). Fcgamma receptors: old friends and new family members.. Immunity.

[18] Baudino L, Azeredo da Silveira S, Nakata M, Izui S (2006). Molecular and cellular basis for pathogenicity of autoantibodies: lessons from murine monoclonal autoantibodies.. Springer Semin Immunopathol.

[19] Yamanaka A, Kosugi S, Konishi E (2008). Infection-enhancing and –neutralizing activities of mouse monoclonal antibodies against dengue type 2 and 4 viruses are controlled by complement levels.. J Virol.

[20] Schlesinger JJ, Foltzer M, Chapman S (1993). The Fc portion of antibody to yellow fever virus NS1 is a determinant of protection against YF encephalitis in mice.. Virology.

[21] Jacobs SC, Stephenson JR, Wilkinson GW (1994). Protection elicited by a replicon-defective adenovirus vector expressing the tick-borne encephalitis non-structural glycoprotein NS1.. J Gen Virol.

[22] Stephenson JR (2005). Understanding dengue pathogenesis: implications for vaccine design.. Bull World Health Organ.

[23] Falconar AKI (2008). Monoclonal antibodies that bind to common epitopes on the dengue virus type 2 nonstructural-1 and envelope glycoproteins display weak neutralising activity and differentiated responses to virulent strains: implications for pathogenesis and vaccines.. Clin Vaccine Immunol.

[24] Falconar AKI (1997). The dengue virus non-structural-1 protein (NS1) generates antibodies to common epitopes on human blood clotting, integrin/adhesion proteins and binds to human endothelial cells: potential implications in haemorrhagic fever pathogenesis.. Arch Virol.

[25] Falconar AKI (2007). Antibody responses are generated to immunodominant ELK/KLE-type motifs on the dengue virus non-structural-1 glycoprotein during live dengue virus infections in mice and humans: implications for diagnosis, pathogenesis, and vaccine design.. Clin Vaccine Immunol.

[26] Innis, BL, Porterfield JS (1995). Dengue and dengue hemorrhagic fever. KASS handbook of infectious diseases: Exotic viral infections.

[27] Jessie K, Fong MY, Devi S, Lam SK, Wong KT (2004). Localisation of dengue virus in naturally infected human tissues by immunohistochemistry and in situ hybridization.. J Infect Dis.

[28] Bhoopat L, Bhamarapravati N, Attasiri C, Yoksarn S, Chaiwun B (1996). Immunohistochemical characterization of a new monoclonal antibody reactive with dengue virus-infected cells in frozen tissue using immunoperoxidase technique.. Asian Pac J Allergy Immunol.

[29] Miagostovich MP, Ramos RG, Nicol AF, Nogueira RM, Cuzzi-Maya T (1997). Retrospective study on dengue fatal cases.. Clin Neuropathol.

[30] Balsitis SJ, Coloma J, Castro G, Alava A, Flores D (2009). Tropism of dengue virus in mice and humans defined by viral nonstructural protein 3-specific immunostaining.. Am J Trop Med Hyg.

[31] Ramos C, Sánchez G, Pando RH, Baquera J, Hernández D (1998). Dengue virus in the brain of a fatal case of hemorrhagic dengue fever.. J Neurovirol.

[32] Blackley S, Kou Z, Chen H, Quinn M, Rose RC (2007). Primary human splenic macrophages, but not T or B cells, are the principal target cells for dengue virus infection in vitro.. J Virol.

[33] Huerre MR, Lan NT, Marianneau P, Hue NB, Khun H (2001). Liver histopathology and biological correlates in five cases of fatal dengue fever in Vietnamese children.. Virchows Arch.

[34] de Macedo FC, Nichol AF, Cooper LD, Yearsley M, Pires AR (2006). Histological, viral, and molecular correlates of dengue fever infection of the liver using highly sensitive immunohistochemistry.. Diagn Mol Pathol.

[35] Quaresma JA, Barros VL, Pagliari C, Fernandes ER, Guedes F (2006). Revisiting the liver in human yellow fever: virus-induced apoptosis in hepatocytes associated with TGF-β, TNF-α and NK cells activity.. Virology.

[36] Lum LC, Thong MK, Cheah YK, Lam SK (1995). Dengue-associated adult respiratory distress syndrome.. Ann Trop Paediatr.

[37] Thong MK (1998). Dengue shock syndrome and acute respiratory distress syndrome.. Lancet.

[38] Kamath SR, Ranit S (2006). Clinical features, complications and atypical manifestations of children with severe forms of dengue hemorrhagic fever in South India.. Indian J Pediatr.

[39] Ong A, Sandar M, Chen MI, Sin LY (2007). Fatal dengue hemorrhagic fever in adults during a dengue epidemic in Singapore.. Int J Infect Dis.

[40] Malavige GN, Ranatunga PK, Jayaratne SD, Wijersiriwardana B, Seneviratne SL (2007). Dengue viral infections as a cause of encephalopathy.. Indian J Med Microbiol.

[41] Wasay M, Channa R, Jumani M, Shabbir G, Azeemuddin M (2008). Encephalitis and myelitis association with dengue viral infection clinical and neuroimaging features.. Clin Neurol Neurosurg.

[42] Soares CN, Cabral-Castro MJ, Peralta JM, de Freitas MR, Zalis M (2011). Review of the etiologies of viral meningitis and encephalitis in a dengue endemic region.. J Neurol Sci.

[43] Jackson ST, Mullings A, Bennett F, Khan C, Gordon-Strachan G (2008). Dengue infections presenting with neurological manifestations in a dengue endemic population.. West Indian Medical J.

[44] Garcia-Rivera EJ, Rigau-Perez JG (2002). Encephalitis and dengue.. Lancet.

[45] Le VT, Phan TQ, Do QH, Nguyen BH, Lam QB (2010). Viral etiology of encephalitis in children in southern Vietnam: results of a one-year prospective descriptive study.. PLoS Negl Trop Dis.

[46] Rothman AL, Gubler DJ, Kuno G (1997). Viral pathogenesis of dengue infections.. Dengue and dengue hemorrhagic fever.

[47] Schlesinger JJ, Bradriss MW, Walsh EE (1987). Protection of mice against dengue 2 virus encephalitis by immunization with the dengue virus non-structural glycoprotein NS1.. J Gen Virol.

[48] Henchal EA, Henchal LS, Schlesinger JJ (1988). Synergistic interactions of anti-NS1 monoclonal antibodies protect passively immunized mice from lethal challenge with dengue 2 virus..

[49] Falgout B, Bray M, Schlesinger JJ, Lai CJ (1990). Immunization of mice with recombinant vaccinia virus expressing authentic dengue virus non-structural protein NS1 protects against lethal dengue virus encephalitis.. J Virol.

[50] Costa SM, Freire MS, Alves AM (2006). DNA vaccine against the non-structural 1 protein (NS1) of dengue 2 virus.. Vaccine.

[51] Lazo L, Hermida L, Zulueta A, Sánchez J, López C (2007). A recombinant capsid protein from dengue-2 induces protection in mice against homologous virus.. Vaccine.

[52] Gil L, López C, Lazo L, Valdés I, Marcos E (2009). Recombinant nucleocapsid-like particles from dengue-2 virus induce protection CD4+ and CD8+ cells against virus encephalitis in mice.. Int Immunol.

[53] Churdboonchart V, Khemarunmanus M, Yoksan S, Bhamarapravati N (1990). Dengue virus serotypic identification using suckling mouse and western blot technique.. Southeast Asian J Trop Med Public Health.

[54] Lee YR, Huang KJ, Lei HY, Chen SH, Lin YS (2005). Suckling mice were used to detect infectious dengue-2 viruses by intracerebral injection of the full-length RNA transcript.. Intervirology.

[55] Leitmeyer KC, Vaughn DW, Watts DM, Salas R, Villalobos I (1999). Dengue virus structural differences that correlate with pathogenesis.. J Virol.

[56] Falconar AKI (1999). Identification of an epitope on the dengue virus membrane (M) protein defined by cross-reactive monoclonal antibodies: design of an improved epitope sequence based on common determinants present in both envelope (E and M) proteins.. Arch Virol.

[57] Avirutnan P, Zhang L, Punyadee N, Manuyakorn, A, Puttikhunt C (2007). Secreted NS1 of dengue virus attaches to the surface of cells via interactions with heparin sulfate and chondroitin sulfate E. PLOS Pathogens.

[58] Flamand M, Megret F, Mathieu M, Lepault J, Rey FA (1999). Dengue virus type 1 nonstructural glycoprotein NS1 is secreted from mammalian cells as a soluble hexamer in a glycosylation-dependent fashion.. J Virol.

[59] Basílio-de-Olivera CA, Aguiar GR, Baldanza MS, Barth OM, Eyer-Silva WA (2005). Pathologic study of a fatal case of dengue-3 virus infection in Rio de Janeiro, Brazil.. Braz J Infect Dis.

[60] Hauser W, Knobeloch KP, Eigenthaler M, Gambaryan S, Krenn V (1999). Megakaryocyte hyperplasia and enhanced agonist-induced platelet activation in vasodilator-stimulated phosphoprotein knockout mice.. Proc Natl Acad Sci USA.

[61] Hall WC, Crowell TP, Watts DM, Barros VL, Kruger H (1991). Demonstration of yellow fever and dengue antigens in formalin-fixed paraffin-embedded human live by immunohistochemical analysis.. Am J Trop Med Hyg.

[62] Hayes EB, Sejvar JJ, Zaki SR, Lanciotti RS, Bode AV (2005). Virology, pathology, and clinical manifestations of West Nile virus disease.. Emerg Infect Dis.

[63] German AC, Myint KS, Mai NT, Pomeroy I, Phu NH (2006). A preliminary neuropathological study of Japanese encephalitis in humans and a mouse model.. Trans R Soc Trop Med Hyg.

[64] Falconar AK, de Plata E, Romero-Vivas CM (2006). Altered enzyme-linked immunosorbent assay immunoglobulin M (IgM)/IgG optical density ratios can correctly classify all primary or secondary dengue virus infections 1 day after the onset of symptoms, when all of the viruses can be isolated.. Clin Vaccine Immunol.

[65] Huang KJ, Yang YC, Lin YS, Huang JH, Liu HS (2006). The dual-specific binding of dengue virus and target cells for the antibody-dependent enhancement of dengue virus infection.. J Immunol.

[66] Mariani M, Camagna M, Tarditi L, Seccamani E (1991). A new enzymatic method to obtain high-yield F(ab)2 suitable for clinical use from mouse IgGl.. Mol Immunol.

[67] Andrew SM, Titus JA (2001). Fragmentation of immunoglobulin G. Curr Protoc Immunol Chapter 2: Unit.

[68] Song X, Shapiro S, Goldman, DL, Casadevall A, Scharff M (2002). Fcgamma receptor I- and III-mediated macrophage inflammatory protein 1 alpha induction in primary human and murine microglia.. Infect Immun.

[69] Quan Y, Möller T, Weinstein JR (2009). Regulation of Fc gamma receptors and immunoglobulin G-mediated phagocytosis in mouse microglia.. Neurosci Lett.

[70] Dheen ST, Kaur C, Ling EA (2007). Microglial activation and its implications in brain diseases.. Curr Med Chem.

[71] Gulati S, Mareshwari A (2007). Atypical manifestations of dengue.. Trop Med Int Health.

[72] Hathirat P, Isarangkura, Srichaikul T, Suvatte V, Mitrakul C (1993). Abnormal hemostasis in dengue hemorrhagic fever.. Southeast Asian J Trop Med Public Heath.

[73] Srichaikul T, Nimmannitya S (2000). Haematology in dengue and dengue haemorrhagic fever. Baillieres Best Pract Res Clin Haematol..

[74] Halstead SB, Feigin RD (2004). Dengue and dengue hemorrhagic fever.. Textbook of pediatric infectious diseases.

[75] Barth OM, Barreto DF, Paes MV, Takiya CM, Pinhao AT (2006). Morphological studies in a model of dengue-2 virus infection in mice.. Mem Inst Oswaldo Cruz.

[76] Bosma KJ, Lewis JF (2007). Emerging therapies for treatment of acute lung injury and acute respiratory distress syndrome.. Expert Opin Emerg Drugs.

[77] Raghavendran K, Pryhuber GS, Chess PR, Davidson BA, Knight, PR (2008). Pharmacotherapy of acute lung injury and acute respiratory distress syndrome.. Curr Med Chem.

[78] Huang S, Hendricks W, Althage A, Hemmi S, Bluethmann H (1993). Immune response in mice that lack the interferon-gamma receptor.. Science.

[79] Johnson AJ, Roehrig JT (1999). New mouse model for dengue virus vaccine testing.. J Virol.

[80] Shresta S, Sharar KL, Prigozhin DM, Beatty PR, Harris E (2006). Murine model for dengue virus-induced lethal disease with increased vascular permeability.. J Virol.

[81] Tan GK, Ng JK, Trasti SL, Schul W, Yip G (2010). A non mouse-adapted dengue virus strain as a new model of severe dengue infection in AG129 mice.. PLoS Negl Trop Dis.

[82] Balsitis SJ, Williams KL, Lachica R, Flores D, Kyle JL (2010). Lethal antibody enhancement of dengue disease in mice is prevented by Fc modification.. PLOS Pathogens.

[83] Schroder K, Hertzog PJ, Ravasi T, Hume DA (2004). Interferon-gamma: an overview of signals, mechanisms and functions.. J Leukoc Biol.

[84] Bozza FA, Cruz OG, Zagne SM, Azeredo EL, Nogueira RM (2008). Multiplex cytokine profile from dengue patients: MIP-1beta and IFN-gamma as predictive factors for severity.. BMC Infect Dis.

[85] Durbin AP, Vargas MJ, Wanionek K, Hammond SN, Gordon A (2008). Phenotyping of peripheral blood mononuclear cells during acute dengue illness demonstrates infection and increased activation of monocytes in severe cases compared to classical dengue fever.. Virology.

[86] Shresta S, Kyle JL, Robert Beatty P, Harris E (2004). Early activation of natural killer and B cells in response to primary dengue virus infection in A/J mice.. Virology.

[87] Schul W, Liu W, Xu HY, Flamand M, Vasudevan SG (2007). A dengue fever viremia model in mice shows reduction in viral replication and suppression of the inflammatory response after treatment with antiviral drugs.. J Infect Dis.

[88] Prestwood TR, Prigozhin DM, Sharar KL, Zellweger RM, Shresta S (2008). A mouse-passaged dengue virus strain with reduced affinity for heparin sulphate causes severe disease in mice by establishing increased systemic viral loads.. J Virol.

[89] Sittisombut N, Sistayanarain A, Cardosa MJ, Salminen M, Damrongdachakul S (1997). Possible occurrence of a genetic bottleneck in dengue serotype 2 viruses between the 1980 and 1987 epidemic seasons in Bangkok, Thailand.. Am J Trop Med Hyg.

[90] Worobey M, Rambaut A, Holmes EC (1999). Widespread intra-serotype recombination in natural populations of dengue virus.. Proc Natl Acad Sci USA.

[91] Chen SP, Yu M, Jiang T, Deng YQ, Qin CF (2008). Identification of a recombinant dengue virus type 1 with 3 recombination regions in natural populations in Guangdong province, China.. Arch Virol.

[92] Perez-Ramirez G, Diaz-Badillo A, Camacho-Nuez M, Cisneros A, de Lourdes Munoz M (2009). Multiple recombinations in two dengue virus, serotype-2 isolates from patients from Oaxaca, Mexico.. BMC Microbiol.

