# Supplementary figures and images for: The NS1 Glycoprotein Can Generate Dramatic Antibody-Enhanced Dengue Viral Replication in Normal Out-Bred Mice Resulting in Lethal Multi-Organ Disease
Source: PLoS One. 2011 Jun 22;6(6):e21024. doi: 10.1371/journal.pone.0021024 (PMC3120820; doi:10.1371/journal.pone.0021024)

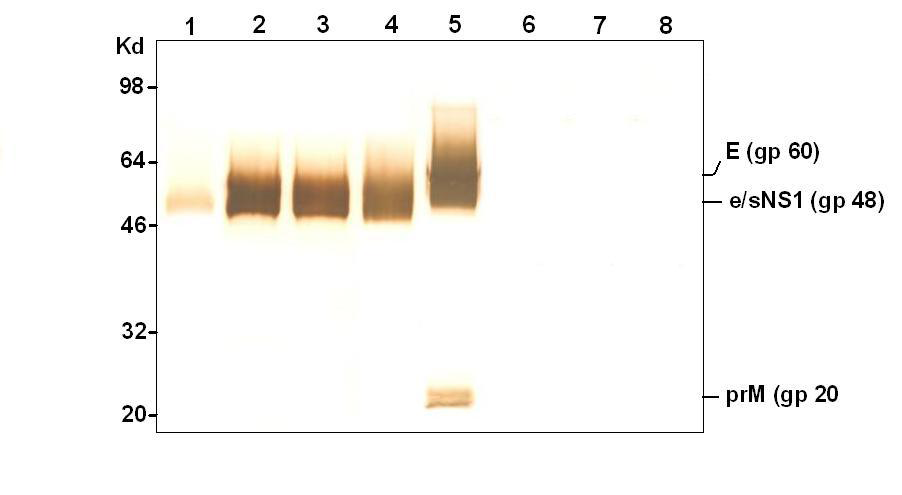

Supplement: Figure S1 — Immunoblot reactions of immuno-affinity purified NS1 glycoproteins. High (960 ng) concentrations of purified DENV-2 (NSx strain) virions (lanes 1 and 5) and high (200 ng) concentrations of the purified e/sNS1 glycoproteins of the 16681 (lanes 2 and 6), NG-C (lanes 3 and 7) and NSx (lanes 4 and 8) were heated at 100°C for 3 min and subjected to 8% (wt/vol) non-reduced SDS-PAGE and immuno-blotting. These strips were then reacted with 1 µg/ml concentrations of MAbs specific for either the DENV NS1 (MAb 2A5.1) glycoprotein (lanes 1 to 4) or the E (MAb 2C5.1) and prM (MAb 2A4.1) (lanes 5 to 8) glycoproteins. The bound MAbs were then detected using peroxidise-labelled anti-mouse IgG2a subclass-specific secondary PAbs and CND substrate. The location of the standard kD molecular weight markers and the DENV-2 E (gp60/55), e/sNS1 (gp48) and prM (gp20) glycoproteins are shown. (TIF) [file pone.0021024.s001.tif]
